# FGF Signaling in Lung Development and Disease: Human Versus Mouse

**DOI:** 10.3389/fgene.2019.00170

**Published:** 2019-03-12

**Authors:** Soula Danopoulos, Jessica Shiosaki, Denise Al Alam

**Affiliations:** Saban Research Institute, Children’s Hospital Los Angeles, Los Angeles, CA, United States

**Keywords:** FGF10, human lung, development, disease, FGF signaling

## Abstract

Fibroblast growth factor 10 (FGF10) plays an important role in mouse lung development, injury, and repair. It is considered the main morphogen driving lung branching morphogenesis in rodents. While many studies have found FGF10 SNPs associated with COPD and branch variants in COPD smokers, there is no evidence of a causative role for FGF10 or these SNPs in human lung development and pediatric lung diseases. We and others have shown divergent roles for FGF10 in mouse lung development and early human lung development. Herein, we only review the existing literature on FGF signaling in human lung development and pediatric human lung diseases, comparing what is known in mouse lung to that in human lung.

## Introduction

The mammalian lung is derived from invagination of the foregut endoderm that ultimately forms the primitive lung buds. These buds undergo a series of branching, proliferation, and differentiation to eventually become a fully functioning air exchange organ. Stages of lung development are thought to be comparable among mammalian species, primarily between humans and rodents, thus making rodents a very widely used model to study lung development and disease. The knowledge gained from rodent studies has been crucial in advancing our understanding of basic biological processes in the lung. However, in light of the low success rate in clinical trials [14% reported in 2018 (Wong et al., 2018)], the necessity to better understand human lung biology has become increasingly important.

**Figure 1:**
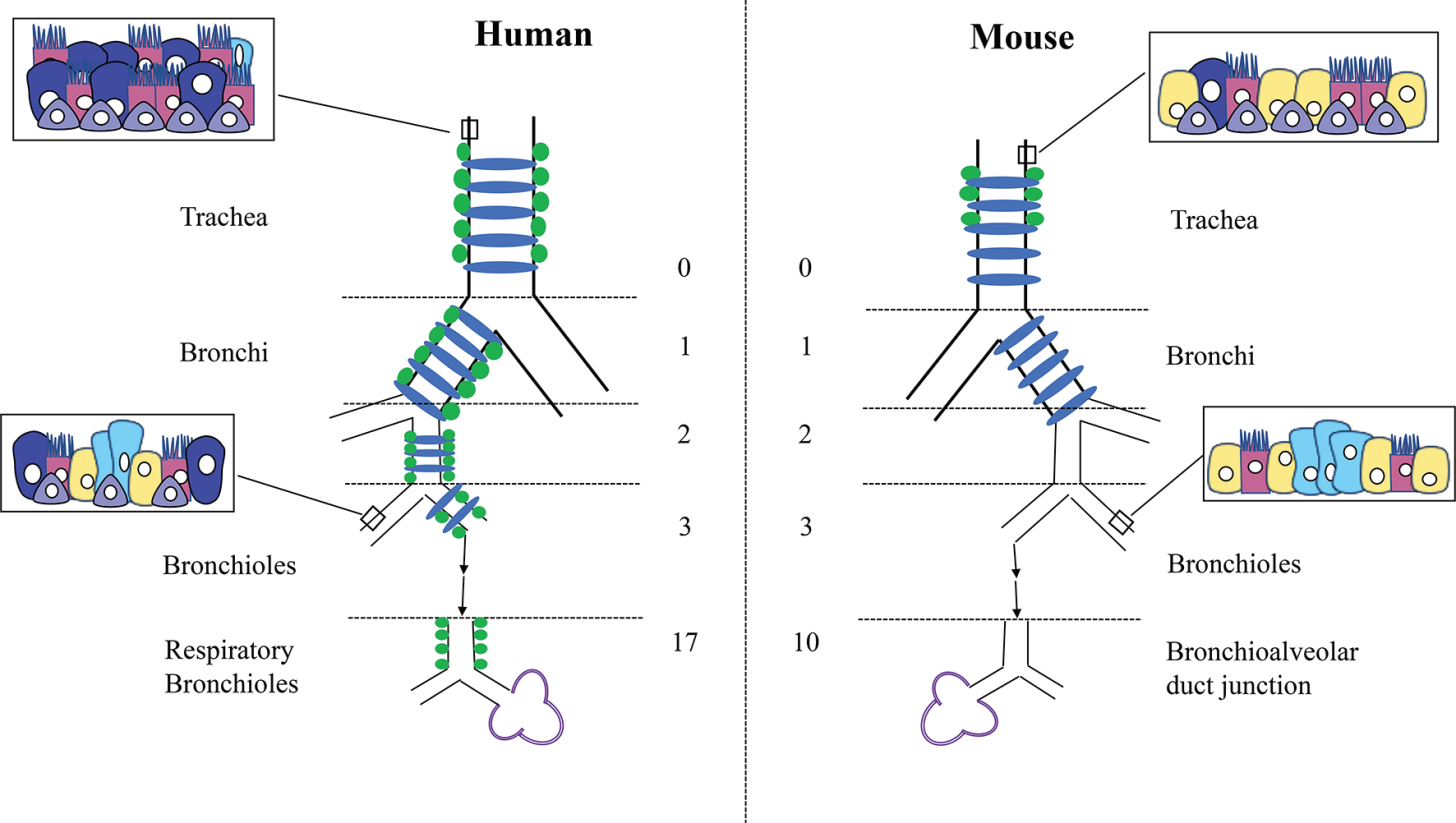
Differences in structure and cellular composition between human and mouse lungs. Cartilage rings (blue) extend into the bronchioles in human lungs but are present only in the trachea and bronchi in mouse. Submucosal glands (green) are limited to the trachea in mouse but are present into the smaller respiratory bronchioles in humans. In mouse, only the trachea and main stem bronchi are lined with pseudostratified epithelium, whereas in humans, all the conducting airways from the trachea to the bronchioles are lined with pseudostratified epithelium. Basal cells (purple) are found deeper into the bronchioles only in humans. The human lung consists of more goblet cells (dark blue) in the proximal epithelium, with Club cells (yellow) mostly restricted to the smaller airways. Conversely, the mouse lung contains more Club cells (yellow) through the trachea and bronchi, with less goblet cells throughout. Neuroendocrine cells (light blue) are present within the bronchioles in clusters in the mouse and isolated or in much smaller clusters throughout the entire epithelial tree in human. Club cells = yellow, Ciliated cells = pink, Basal cells = purple, Goblet cells = dark blue, Neuroendocrine cells = light blue.

Although there are numerous similarities between the mouse and human lung, many differences have also been noted at the structural, cellular, and molecular levels. Grossly, the structure, size, and scale of the lung are notably different between the two species. Although they are both five lobed units, the mouse lung comprises a single large left lobe and four right lobes (cranial lobe, middle lobe, caudal lobe, and accessory lobe), whereas the human lung has two left lobes (superior lobe of left lung and inferior lobe of left lung) and three right lobes (superior lobe of right lung, middle lobe of right lung, and inferior lobe of right lung). Embedded in these lobes is the highly structured, arborized, epithelial tree. Starting from the trachea as generation 0 and moving down to the bronchioles, the human lung contains a total of 17–21 branch generations, whereas in mouse, there are only 13–17 branch generations (Irvin and Bates, 2003). This also reflects differences in total lung capacity, alveolar numbers and volume. The total lung capacity is 6 × 10^3^ ml in adult humans compared to 1 ml in adult mouse (Irvin and Bates, 2003). The volume of one alveolus is 4.2 × 10^6^ μm^3^ in humans compared to 2.2 × 10^4^ μm^3^ in mouse (Wansleeben et al., 2013), and the estimated average number of alveoli is 4.8 × 10^8^ in humans compared to 2.31 × 10^6^ in mouse (Ochs et al., 2004; Knust et al., 2009). In addition to the very apparent differences in size and structure, there have also been multiple cellular and molecular differences that have been described during development and adulthood. For instance, cartilaginous rings are restricted to the trachea and main bronchi in mouse, whereas in humans, these extend into the bronchioles (Figure 1). Similarly, submucosal glands are only present in the upper part of the mouse trachea, whereas in humans, they extend further down into the respiratory bronchioles. Differences in epithelial structure and cell type distribution are further detailed in Figure 1. These changes in cellular composition result in different molecular interactions and expression patterns.

**Figure 2:**
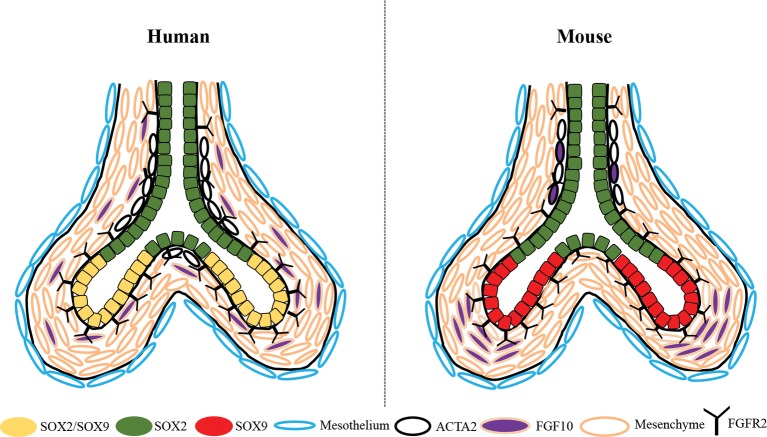
Schematic depiction of the differential expression of SOX2, SOX9, ACTA2, and FGF10 between the developing human and mouse lung during the pseudoglandular stage. SOX2/SOX9 double positive progenitor cells are present in the distal epithelial tips of the human lung, whereas only SOX9+ cells are present in the distal tips of the mouse. ACTA2+ smooth muscle cells are found in both human and mouse surrounding the proximal airway, but extend more distally into the human lung and are closely associated with SOX2+ cells only, they are also in human at the bifurcation of the branch tips. In mouse lung, *Fgf10* is highly expressed in the mesenchyme adjacent to the epithelial tips and in the smooth muscle cells surrounding the airways; whereas in humans *FGF10+* cells are found dispersed throughout the mesenchyme with little expression in the smooth muscle cells.

For years, it was well-established that the progenitor cell population of the proximal epithelium in the developing mouse lung is Sox2+, whereas that of the distal epithelium is solely Sox9+. We and others have demonstrated that during the pseudoglandular stage of human lung development, the distal epithelial cells express a double positive SOX2/SOX9 progenitor cell population that is no longer present during the canalicular stage of development, suggesting its importance to human lung branching morphogenesis (Figure 2) (Nikolic et al., 2017; Miller et al., 2018; Danopoulos et al., 2018a). This difference of progenitor cell populations is accompanied by a change in smooth muscle cell expression. In mouse, ACTA2+ cells are primarily localized in the proximal region of the epithelium, surrounding the Sox2+ cells, whereas in humans, we showed that the ACTA2+ cells extend to the most distal region of the developing lung, surrounding the SOX2+ cells of the proximal epithelium, as well as being located between the clefts of branch points, yet not extending into the region of the SOX2/SOX9 population (Figure 2) (Danopoulos et al., 2018a). Nikolic et al. performed transcriptomic analyses of these SOX2/SOX9 positive cells as well as SOX2 stalk cells and compared them to available transcriptomic data from mouse lung epithelial tips. The authors showed that the transcriptomic profile of these SOX2/SOX9 epithelial tips shares similarities with mouse epithelial tips, but more importantly, they showed that some genes were unique to mouse and others were unique to humans, claiming about 348 total unique genes in human lung tips (Nikolic et al., 2017). These analyses were performed only in epithelial lung tip cells, excluding the many other cell types in the lung. Similar comparison and observations were also made by Miller et al. (2018). Many of these molecular and cellular differences between mouse and human lung development are of great significance and may pertain to lung branching morphogenesis such as the SOX2/SOX9 double positive population shown to be required for proper lung branching (Danopoulos et al., 2018a).

Recent advances in human lung development and the *in vitro* models used for these studies have been recently reviewed by Nikolic et al. (2018). Other reviews by Prince and Yuan et al. have also extensively reviewed the role of FGF10 in lung development, homeostasis, and disease, across the life span and in animal models (Prince, 2018; Yuan et al., 2018). In the present concise review, we mainly focus on FGF signaling during human lung development and congenital/pediatric human lung diseases.

## FGF Family

Fibroblast growth factors are a family of growth factors described in many multicellular species such as xenopus, drosophila, zebrafish, rodents, and humans and are known to be involved in a wide range of biological processes, including organogenesis, homeostasis, repair, and metabolism. There are 22 known FGFs in both human and mouse that have been divided into seven different subfamilies (FGF1, FGF4, FGF7, FGF8, FGF9, FGF11, and FGF15/19), which are categorized according to similarities in biochemical function, sequence, and evolutionary relationships (Ornitz and Itoh, 2015). The FGF1, FGF4, FGF7, FGF8, and FGF9 subfamilies are known as the canonical FGFs, suggesting that they function in a paracrine/autocrine manner, binding to one of the FGFRs (FGFR1-FGFR4, with FGFR1-FGFR3 having a IIIb or IIIc splice variant), with heparin or heparan sulfate as a cofactor (Ornitz and Itoh, 2015). This in turn activates phosphorylation of a specific tyrosine residue on the FGFR, resulting in the initiation of downstream intracellular signaling pathway: RAS-MAPK, PI3K-AKT, PLCγ, or STAT. FGF10 is a canonical FGF and belongs to the FGF7 family (Furdui et al., 2006; Ornitz and Itoh, 2015). The FGF11 subfamily, consisting of FGF11, FGF12, FGF13, and FGF14, is categorized as the intracellular/intracrine FGFs, which as a result do not bind to any of the FGFRs (Olsen et al., 2003). The members of this subfamily are associated with voltage-gated sodium channels and the regulation of neuron activity. The FGFs associated in the final subfamily (FGF15/19, FGF21, and FGF23), the FGF15/19, are also known as the endocrine FGFs. This is where human and mouse differ in terms of FGF expression. In humans, there is no Fgf15, whereas in rodents (mice and rats), there is no expression of FGF19. Therefore, it is believed that Fgf15 and FGF19 are orthologous genes (Wright et al., 2004). The endocrine FGFs do bind to FGFRs; however, in order to increase the affinity to the receptor, it is necessary to have the presence of an additional single pass transmembrane protein known as Klotho (alpha or beta) to act as a cofactor and ensure binding efficiency (Hu et al., 2013). With the presence of 22 different ligands and 4 receptors, not including the different isoforms, it is quite evident that FGF signaling is very complex.

## FGFS and FGFRS Mutations are Associated with Human Lung Diseases

Although there is a clear association between *Fgf10* mutations and lung malformation in mouse, such is not evident in humans. The two most familiar conditions associated with human *FGF10* mutations are autosomal dominant aplasia of lacrimal and salivary glands (ALSG) (Entesarian et al., 2005; Seymen et al., 2017) and lacrimo-auriculo-dento-digital syndrome (LADD) (Milunsky et al., 2006; Rohmann et al., 2006), with both conditions lacking a primary lung complication. LADD patients also harbor mutations in the *FGFR2* and *FGFR3* genes (Rohmann et al., 2006), suggesting that additional FGFs may be contributing factors to the syndrome. Although one study demonstrated that ALSG patients displayed lower lung function (reduced FEV1) and irreversible airway obstruction consistent with chronic obstructive pulmonary disease (COPD) (Klar et al., 2011), 6 of the 12 patients studied had asthma and another four had other allergies. Therefore, it is unclear whether lung function changes were a direct cause of *FGF10* mutations or other unrelated factors. Furthermore, none of these patients were diagnosed with lung malformation (Klar et al., 2011). Another study suggested that single nucleotide polymorphisms (SNPs) in the *FGF10* gene may be associated with COPD (Ren et al., 2013). Interestingly, it has also been shown that *FGF10* SNPs are associated with airway branch variants, where the right medial basal segmental airway was absent in smoker COPD patients but remained unaffected in nonsmoker COPD patients (Smith et al., 2018). In addition, significant associations between lung function and SNPs in the FGF10 gene have been found (Jackson et al., 2018). However, combined, these studies still do not establish a direct causative role for *FGF10* mutations/SNPs in human congenital lung malformations, specifically in branching defects. Importantly, the effect of these SNPs on *FGF10* gene expression is unclear. A recent study by Karolak et al. demonstrated the presence of rare FGF10 and TBX4 mutations in lethal pulmonary acinar dyslasia and alveolar dysplasia (Karolak et al., 2019). They showed that FGF10 mutations or SNPs are mostly associated with alveolar dysplasia occurring past the pseudoglandular stage of development. However, the study suggested that these mutations alone are not sufficient to cause these severe lung phenotypes but rather support complex compound inheritance of additional noncoding variants or a genetic modifiers other places in the genome (Karolak et al., 2019). Finally, one cannot exclude that homozygous mutations in FGF10 might cause yet undocumented lethality in early fetal life.

Alternatively, FGF receptors have been associated with lung or airway anomalies. Activating mutations of FGFR2 cause Crouzon, Apert, and Pfeiffer syndromes (Robertson et al., 1998). While these syndromes are primarily characterized by craniofacial and skeletal defects, defects in tracheal cartilaginous ring formation resulting in mortality due to respiratory distress have also been reported (Devine et al., 1984; Cinalli et al., 1995; Gonzales et al., 2005). In these patients, the only lung associated deficiency witnessed is a cartilaginous sleeve that surrounds the trachea with no visible cartilage rings. In contrast, homozygous FGFR2 loss of function mutation (p.R255Q) results in ectrodactyly and pulmonary acinar dysplasia, a rare congenital lung malformation characterized by in utero arrest of lung development at the pseudoglandular stage (Barnett et al., 2016). This suggests that *FGFR2* signaling plays a key role in the progression of lung development from the pseudoglandular stage onwards. However, it is unclear which *FGFR2* ligands are responsible for this effect.

Moreover, SNPs in the *FGFR4* gene were shown to be associated with bronchopulmonary dysplasia (BPD) and respiratory distress syndrome (RDS), whereas SNPs in the *FGFR2* gene had no association with neither BPD nor RDS (Rezvani et al., 2013). SNPs in *FGF3* and *FGF7* but not *FGF2*, *FGF4*, or *FGF18* showed associations with RDS only but not BPD (Rezvani et al., 2013). These findings suggest *FGFR4, FGF3*, *and FGF7* play an important role in human distal lung growth.

## FGF10 in Human Lung Development

Several FGF ligands and receptors have been shown to play important roles in organogenesis of multiple organ systems including the lung. In particular, *Fgf10* has been shown to be the main morphogen driving lung branching morphogenesis in mouse. Absence of *Fgf10* or its receptor *Fgfr2b* results in complete lung agenesis in mouse (Sekine et al., 1999; De Moerlooze et al., 2000). Until recently, the temporal and spatial expression of *FGF10* and its receptors in the developing human lung were unknown. We showed that *FGF10* is expressed throughout human lung development from 10 weeks of gestation up to 21 weeks of gestation (Al Alam et al., 2015; Danopoulos et al., 2018b). Unlike mouse where *Fgf10* expression increases during the pseudoglandular stage, in humans, *FGF10* expression was stable in the pseudoglandular stage and increased significantly in the canalicular stage (Al Alam et al., 2015; Danopoulos et al., 2018b). Fluorescent *in situ* hybridization allowed us to assess the spatial distribution of *FGF10* in the developing human lung and we showed that *FGF10* is expressed throughout the lung parenchyma with some expression in the airway and vascular smooth muscle cells (Danopoulos et al., 2018b). This expression was different than what has been described in mouse where *Fgf10* is abundantly expressed in the distal mesenchyme adjacent to epithelial buds (Bellusci et al., 1997). FGF10 receptors, *FGFR1* and *FGFR2*, are expressed in both epithelium and mesenchyme of the human developing lung between 11–18 weeks of gestation (Danopoulos et al., 2018b) with a stronger expression of *FGFR2* in the distal epithelium as compared to proximal epithelium, comparable to what is observed in mouse. The importance of FGF10 localization for directed branching has been reported in several mouse studies (Bellusci et al., 1997; Hirashima et al., 2009; Makarenkova et al., 2009) though others suggest that this localization is not important for the branching process (Volckaert et al., 2013).

The role of FGF10 in human lung development was unexplored until recently, and it remains incompletely understood. A report by Graeff et al. in 1999 showed that FGF7 and FGF10 both induced liquid secretion and enlargement in distal tips in human fetal lung explants cultured *in vitro* (Graeff et al., 1999). In mouse lung explants in vitro, FGF10 induces branch formation and allows the maintenance of the proximal-distal patterning characterized by SOX2 expression proximally and SOX9 expression distally (Volckaert et al., 2013). We performed more in depth analyses of the effect of FGF10, but also FGF7 and FGF9 on human lung explants cultured in vitro. We demonstrated, similar to the previous work, that FGF10 induces distal bud cysting and inhibits branching in human lung explants in vitro (Danopoulos et al., 2018b). Moreover, FGF10 does not modulate proliferation of either the epithelium or the mesenchyme following 48 hours culture in vitro (Danopoulos et al., 2018b); though an increase in p-ERK was seen, demonstrating that FGF10 successfully binds to its receptor(s) and induces downstream signaling activation in our culture conditions. Other groups have used in vitro organoid cultures from 12 weeks post conception human fetal lung distal epithelial tips to assess the role of growth factors on growth, branching, and self-renewal (Nikolic et al., 2017; Miller et al., 2018). Miller et al. initially showed that these organoids grow and expand up to 6 weeks in presence of FGF7, CHIR99021 (GSK3 inhibitor), retinoic acid (RA), and FGF10. However, removal of FGF10 alone from the media did not affect the growth or expression of the distal tip markers SOX2 and SOX9, suggesting that FGF10 is dispensable for the maintenance and growth of these human bud tips. Similar results were obtained on organoids derived from human pluripotent stem cells (hPSCs) (Miller et al., 2018). Nikolic et al. grew 5–9 weeks postconception human fetal lung tips with a combination of 7 factors (EGF, FGF7, FGF10, NOGGIN, RSPO1, CHIR99021, and the TGFβ inhibitor SB431542) (Nikolic et al., 2017). They showed that removal of FGF10 from the culture media at day 13 (during the branching period) did not affect the initial establishment of the organoid. Following passaging of the organoids, withdrawal of FGF10 only at day 6 of culture (when organoids are spherical) for 3 days did not alter organoid morphology or RNA expression of *SOX2* and *SOX9*, whereas a decrease in SOX2/SOX9 double positive cells was seen by IF. Taken together, these studies suggest that FGF10 is not required for the initial establishment of SOX2/SOX9 double positive progenitors and for human lung branching, while FGF signaling is important in this process. However, both of these studies used a GSK3 inhibitor CHIR99021 that could possibly compensate for the removal of FGF10, as FGF10 is a major activator of ß-catenin signaling. Interestingly, a more recent study showed that treatment of foregut spheroids with 1% serum and high concentrations of FGF10 was sufficient to the generation of lung organoids containing airway like structures, mesenchymal cells, and cells expressing alveolar epithelial cells type I and type II markers (Miller et al., 2019). But, FGF10 alone was not sufficient to give rise to the bud tip progenitor population co-expressing SOX2 and SOX9. Together, this suggests an important role for FGF10 signaling in later stages of human lung development, more specifically the establishment of the distal fate past the pseudoglandular into canalicular stage and alveolar formation, as well as during the differentiation of airway epithelial cells. Furthermore, it is still unclear whether another FGF ligand or a combination of ligands plays the role in human lung branching that FGF10 plays in mouse lung branching.

## FGF10 in Pediatric Human Lung Diseases

Whereas FGF10 mutations alone may not be sufficient to cause severe human lung malformations or congenital lung disease, alterations in *FGF10* expression have been described in bronchopulmonary dysplasia (BPD). The number of mesenchymal cells staining positive for FGF10 was decreased in the lung of BPD patients as compared to controls (Benjamin et al., 2007). In contrast, two independent GWAS studies found no association between BPD and FGF10 SNPs and no change in *FGF10* gene expression between BPD and controls (Bhattacharya et al., 2012; Li et al., 2015; Hamvas et al., 2018). Additionally, in congenital cystic adenomatoid malformation (CCAM), *FGF10, FGF7*, and *FGFR2* gene levels are unchanged in the lung mesenchyme (Cass et al., 1998; Jancelewicz et al., 2008), whereas epithelial *FGF9* expression is increased 4-fold in the CCAM samples compared to controls (Jancelewicz et al., 2008). Furthermore, there is no evidence of altered *FGF10* expression or signaling in human congenital small lung or lung hypoplasia, although its expression is different in mouse and rat experimental models of hypoplasia (Teramoto et al., 2003; Wang et al., 2018). However, a decrease in *FGF18* expression has been described in hypoplastic lungs from patients with congenital diaphragmatic hernia (Boucherat et al., 2007). In cystic fibrosis, fluid secretion and transport defects play an important role in the pathogenesis of the disease. While there is no clear evidence in human data sets of a role for FGF10 and/or FGF10 signaling in cystic fibrosis, studies in pig models showed that FGF10 treatment induced fluid secretion in non-CF fetal lung explants but was unable to do so in CF fetal lung explants (Meyerholz et al., 2018), suggesting that FGF10 signaling may play an important role in the pathogenesis of the disease.

## Conclusion

The role of FGF10 signaling in human lung development and disease remains poorly understood. Despite the limitations associated with the use and manipulation of human tissues, studies in human development and disease are necessary to better understand the role of FGF10 and other pathways that have been shown to be important in mouse, e.g., FGF, Wnt, and Hippo pathways. The disappointingly high failure rate of clinical trials has made studying human tissues critical, along with the necessity to accelerate the development of therapies for pediatric lung diseases. FGF10, under the drug name Repifermin, failed to prove efficacy in clinical trials for wound healing, mucositis, and ulcerative colitis (Freytes et al., 2004), though it had highly protective and therapeutic effects in animal models. Understanding the role of FGF signaling in human lung development and disease will help tailor its possible therapeutic effects and targets, if any, to the appropriate site of action and patient population.

## Author Contributions

SD, JS and DA reviewed the literature, wrote the review, edited and approved the final version.

### Conflict of Interest Statement

The authors declare that the research was conducted in the absence of any commercial or financial relationships that could be construed as a potential conflict of interest.
